# Menstrual Cycle Modulates Motor Learning and Memory Consolidation in Humans

**DOI:** 10.3390/brainsci10100696

**Published:** 2020-10-01

**Authors:** Koyuki Ikarashi, Daisuke Sato, Kaho Iguchi, Yasuhiro Baba, Koya Yamashiro

**Affiliations:** 1 Field of Health and Sports, Graduate School of Niigata University of Health and Welfare, 1398 Shimami-cho, Kita-Ku, Niigata City 950-3198, Japan; wtm19001@nuhw.ac.jp (K.I.); wtm18001@nuhw.ac.jp (K.I.); 2 Institute for Human Movement and Medical Sciences, Niigata University of Health and Welfare, 1398 Shimami-cho, Kita-Ku, Niigata City 950-3198, Japan; yamashiro@nuhw.ac.jp; 3 Department of Health and Sports, Niigata University of Health and Welfare, 1398 Shimami-cho, Kita-Ku, Niigata City 950-3198, Japan; baba@nuhw.ac.jp

**Keywords:** menstrual cycle, female, motor learning, primary motor cortex

## Abstract

Numerous studies have noted that sex and/or menstrual phase influences cognitive performance (in particular, declarative memory), but the effects on motor learning (ML) and procedural memory/consolidation remain unclear. In order to test the hypothesis that ML differs across menstrual cycle phases, initial ML, overlearning, consolidation, and final performance were assessed in women in the follicular, preovulation and luteal phases. Primary motor cortex (M1) oscillations were assessed neuro-physiologically, and premenstrual syndrome and interoceptive awareness scores were assessed psychologically. We found not only poorer performance gain through initial ML but also lower final performance after overlearning a day and a week later in the luteal group than in the ovulation group. This behavioral difference could be explained by particular premenstrual syndrome symptoms and associated failure of normal M1 excitability in the luteal group. In contrast, the offline effects, i.e., early and late consolidation, did not differ across menstrual cycle phases. These results provide information regarding the best time in which to start learning new sensorimotor skills to achieve expected gains.

## 1. Introduction

The female menstrual cycle is controlled by dynamic periodic and periodic fluctuations in gonadal neuro-steroid hormones. Shifts in the ovarian hormones estradiol and progesterone, as well as their major metabolites, modulate not only neural activity [1,2,3] but also neuroplasticity [4]. Neuroplasticity is the capacity of the nervous system to modify itself, functionally and structurally, in response to experience, injury and intervention [5,6]; this capacity plays an essential role in learning and memory [7,8]. To date, numerous studies have observed that sex differences and/or menstrual cycles influence various types of cognitive performance [9,10]; these studies have especially focused on declarative memory [11,12,13]. On the other hand, the effects of the menstrual cycle on procedural memory with motor learning (ML) and memory consolidation remain unclear. ML is the process by which movements produced alone or in sequence come to be performed effortlessly through repeated practice and interactions with the environment [14]; many skills that are important in daily life (e.g., grasping a glass, using chopsticks, practicing sports, playing a musical instrument) are acquired through ML. Therefore, exploring the interaction of progesterone and estradiol with ML and associated neurophysiological activity is key to understanding how procedural memory and related neural activity change in females across the menstrual cycle. Such research could expand on previous knowledge of the neurophysiological and behavioral changes related to the menstrual cycle in humans.

The gonadal neuro-steroids progesterone and estradiol are both transported to the brain through the blood-brain barrier [15]. Animal studies have shown that estradiol concentrations tend to increase during the post-ovulation stage, compared to preovulation, in the cerebral cortex, as well as in subcortical regions such as the hippocampus, hypothalamus, midbrain, striatum, medulla oblongata, and cerebellum [16]. However, the overall levels are lower, and there are no significant differences in the ratio of estradiol concentration during pre- and post-ovulation in the cerebral cortex. In contrast, the ratio of pre- to post-ovulation progesterone concentrations is much higher in the cerebral cortex than in the other brain regions. Progesterone also has the highest absolute mean concentration in the cerebral cortex. Additionally, these gonadal neuro-steroids have been shown to modulate neuroplasticity in the cerebral cortex. To date, several studies have reported the modulation of neuroplasticity across the menstrual cycle. Noninvasive brain stimulation (NIBS) studies showed that the changes in cortical excitability (e.g., in the primary motor cortex and dorsolateral prefrontal cortex) in response to repetitive transcranial magnetic stimulation (rTMS) and/or transcranial direct current stimulation (tDCS) depended on ovarian hormones [17,18]. These studies reported that increased neural plasticity was at its highest when estrogen concentration was lowest. On the other hand, progesterone seems to have the opposite effect on neural plasticity, in light of another study that found decreasing neural plasticity in the luteal phase in humans [4]. Based on these results, it is expected that ML and memory consolidation, which involve neuroplasticity in the primary motor cortex (M1) [19,20], will be affected by estradiol, but changes may be predominantly driven by progesterone.

Notably, M1 plays a crucial role in ML, as evidenced in several animal studies [21,22,23,24]. In humans, early studies using transcranial magnetic stimulation (TMS) showed that learning various motor tasks was associated with a functional reorganization of M1, as assessed by corticospinal excitability changes [19,25,26], and modulation of M1 excitability by rTMS modified ML in healthy subjects [27,28,29,30]. Additionally, synchronized oscillations in the β and γ frequency bands in M1 are involved in ML. For example, previous studies recording cortical oscillations through various neurophysiological techniques have shown that local changes in both β and γ power influenced various forms of learning [31,32,33,34,35]. These results suggest that either a reduction in β oscillations or an increase in γ activity during training was associated with improved behavioral performance. Recent studies have reported that performance gain during ML and memory consolidation after ML are dependent on M1 oscillatory activity during ML during a transcranial alternating current stimulation (tACS). In particular, increased tonic β oscillatory activity in the M1 interfered with endogenous β rhythms and cortical reorganization associated with ML [36]. For this reason, the amount of β suppression is taken specifically to represent a neurophysiological marker of early cortical reorganization associated with ML [35]. On the other hand, increased β oscillations in M1 immediately after ML facilitated retrieval during the early consolidation phase [37], which was explained by the functional role of β oscillations in maintaining the current motor state [38]. Previous studies have shown that resting β oscillations in M1 reflected neural inhibition [39,40], and transient suppression of neural inhibition is necessary for long-term potentiation (LTP) [41] and ML [42]. It is possible that alterations in neural inhibition induced by fluctuations in progesterone and estradiol concentrations would influence β oscillations in M1 and the performance gain and memory consolidation induced by ML. Additionally, previous studies have reported that neural inhibition in M1 alters across the menstrual cycle due to premenstrual syndrome (PMS), which is defined as the recurrent, cyclical set of emotional and physical symptoms, which occur specifically during the late luteal phase of the menstrual cycle and abate at the onset of menses [43,44,45]. For example, Smith et al. [46] found that the luteal phase along with increased progesterone levels was accompanied by an abnormal M1 excitability (hyper-excitability) in individuals with PMS [47].To date, little is known about the relationship between PMS and ML, although a few studies have reported ML-associated neural activity. Previous results may lead to the hypothesis that PMS-induced abnormal M1 excitability would influence β oscillations and interfere with ML, because neural inhibition has an important role in ML, as previously mentioned.

The present study investigated whether the menstrual cycle of females influences performance gain during ML and memory consolidation after ML and explored its neural mechanisms, with a focus on M1 oscillatory activity. We hypothesized that both the performance gain and memory consolidation of ML would depend on the menstrual cycle. Considering that progesterone has been shown to modulate inhibitory neural circuits [48,49], performance gain during ML would be interfered with by abnormal M1 excitability due to the interaction with gonadal neuro-steroids progesterone and estradiol, and/or by PMS in the luteal phase compared to the follicular and preovulation phases. In contrast to performance gains, early consolidation within a few hours may be facilitated by a progesterone-induced increase in β oscillations during ML. However, these menstrual-induced behavioral, neurophysiological alterations may not carry over to late consolidation (i.e., overnight) and retention (i.e., several days) after the initial ML, because distinct neural mechanisms are involved in the early consolidation that occurs within minutes to hours and in late consolidation and retention that are observed after some days and longer [50]. Late consolidation and retention with sleep involve reorganization over distributed brain circuits, including cortical and subcortical regions (e.g., M1, basal ganglia, cerebellum), unlike performance gain in early consolidation with local plastic change [50,51].

## 2. Materials and Methods

### 2.1. Participants

Forty-two healthy right-handed females aged 20–22 years volunteered to participate in the study. Their handedness was assessed using the Edinburgh Handedness Inventory [52]. The participants were required to have no history of neurological or psychiatric disorders, including premenstrual dysphoric disorder, or any self-reported major menstrual cycle-related changes in mood. They were also required not to smoke and not to be on any prescription medications or hormonal contraceptives. Moreover, they were instructed to avoid alcohol consumption, sleep deprivation and hard physical activity for at least two days before the start of the experiment. The present research was approved by the ethics committee of Niigata University of Health and Welfare, Japan (18354-200122). All experiments conformed to the Declaration of Helsinki. The participants provided informed written consent prior to participation.

### 2.2. Procedures

After being recruited, all participants were required to measure their sublingual temperature every morning immediately after waking up. Then, they were recorded for two menstrual cycles leading up to their first session in order to estimate accurate cycle length. During this phase, 11 participants were excluded from the assignment to groups and analysis because five had irregular menstruation, two began taking pills after starting the experiment, and four could not participate in the measurements due to personal circumstances.

**Figure 1:**
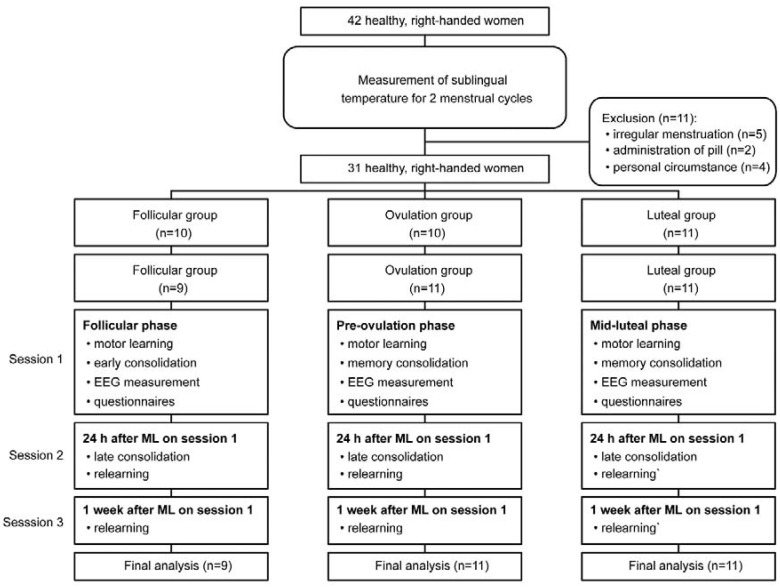
Participant assignment flowchart and overall schedule.

All participants were randomly divided into three groups: follicular (*n* = 10), ovulation (*n* = 10) and luteal (*n* = 11). However, one participant in the follicular group moved to the ovulation group because she could not come to the laboratory on the scheduled day for personal reasons. In the final analysis, we used the data of 9, 11 and 11 participants in the follicular, ovulation and luteal groups, respectively. The participants in each group were required to visit the laboratory in a particular phase of the menstrual cycle: one–two days after menses in the follicular group (low progesterone and estradiol levels), the preovulation phase in the ovulation group (low progesterone and high estradiol level), and the midluteal phase in the luteal group (high progesterone and estradiol levels). All participants performed ML on the appointed day for each group (Session 1), the day after Session 1 (Session 2) and one week after Session 1 (Session 3). All participants performed initial ML in Session 1 from 11:00 to 14:00 to prevent time of day from influencing the results. The flow of participant assignment and overall schedule are shown in Figure 1. The appointment for measurement and the confirmation of each cycle phase were performed according to previous studies [4,53,54]. The initial measurements were scheduled in accordance with each participant’s cycle length, and self-reports, and cycle phases were confirmed by follow-up reports of the next cycle and ovulation tests (FDA-510(K), Doctor’s Choice Ovulations Test, Torrance, CA, USA), which indicated an increase in luteinizing hormone before ovulation [54]. The follicular group was scheduled on the first or second day after menses or as soon as possible thereafter (2.41 ± 0.88 days). The initial measurements in the ovulation group were conducted from the first day to the third day after that day when the ovulation test showed a positive result (2.0 ± 0.82 days). The luteal phase ranged from the third day after ovulation to three days before the onset of the next menses (7.9 ± 3.21 days). To ensure the correct menstrual cycle phase, urinary hormone levels in the three phases were measured in all participants, because there was high interindividual variability in hormone levels. The menstrual cycle phase was additionally assured by urinary hormone levels in each group, and the participants were excluded if the levels were not as expected for both hormonal values.

### 2.3. ML, Consolidation and Retention, and Overlearning

**Figure 2:**
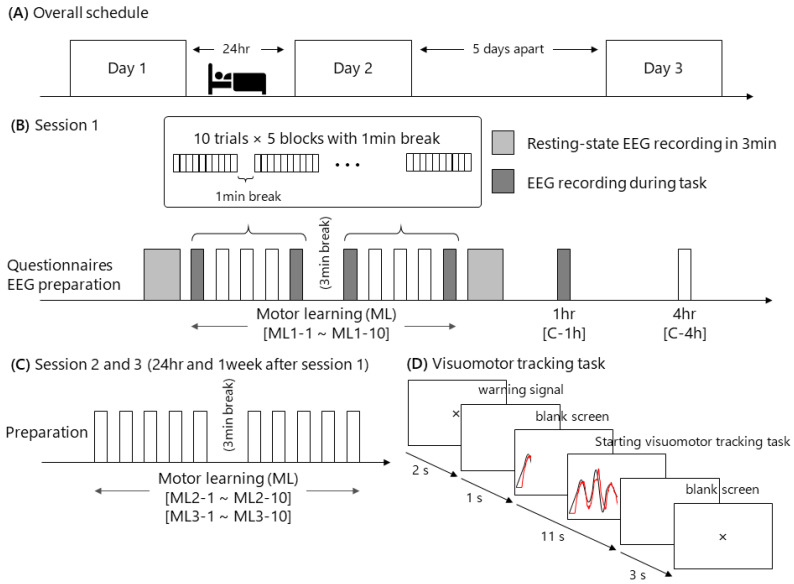
Experimental procedure for motor learning (ML) and overlearning. (**A**) shows the overall schedule of the present experiment. Each participant performed the initial ML on the appointed day (Session 1), followed by overlearning at 24 h (Session 2) and 1 week (Session 3) after the completion of the initial ML. (**B**) presents the experimental procedure during Session 1. After arriving at the laboratory, the participants answered questionnaires about their characteristics, premenstrual syndrome (PMS) and interoceptive awareness, followed by preparation for electroencephalography (EEG) measurements. Next, baseline EEG was recorded in the resting state. Subsequently, the initial ML task, consisting of two sets of 5 blocks with 10 visuomotor tracking task trials in each block (termed ML1-1 to ML1-10), was conducted; EEG recordings were taken during the first, fifth, sixth and tenth blocks (ML1-1, 1-5, 1-6 and 1-10). Resting-state EEG was recorded immediately after the initial ML. Later, one block was performed 1 h after the end of the initial ML with EEG recording, and one block was performed 4 h after the initial ML was completed. (**C**,**D**) show the experimental procedures during Session 2 and Session 3. The participants performed ML with the same sensorimotor tasks as in the initial ML (as overlearning) without EEG recordings. (**D**) shows a paradigm of the present visuomotor tracking task.

The participants conducted ML via a visuomotor tracking task and performed a consolidation test 1 h and 4 h after ML during Session 1; and performed ML (overlearning) at 24 h (Session 2) and 1 week (Session 3) after initial ML (Figure 2) with their dominant hand. After session 1, we measured to assure that the level of consolidation did not change between 1 h and 4 h after initial learning, because previous studies have reported retroactive interference occurring until 4 h after initial ML [55,56]. We used a custom-built PC program (DASYLab version 2016, Measurement Computing Corp.) for the visuomotor tracking task (Figure 2). Each trial lasted 17 s. In each trial, a warning signal and blank screen were presented for 2 and 1 s, respectively. Then, the target black line appeared from the bottom left corner of the monitor and moved to the right side while moving up and down for 11 s. Simultaneously, a red line appeared from the same point as the black line and moved to the right side of the monitor over the course of 11 s. The first 1 s was excluded from the task performance evaluation. A blank screen was then presented for 3 s until the next trial commenced. The participants were instructed to adjust the red line to the target black line on the screen by controlling a force transducer in the participant’s hand. By pinching the force transducer, the participants moved the red line vertically on the screen in real-time and in proportion to the applied force. Increasing pinch force produced upward red line movement, and decreasing pinch force produced downward red line movement. The range of force modulation was 5–15% of the maximal force measured before the ML. The participants were also instructed to relax when not performing the motor task. The present visuo-motor task required a longer time for the execution of one trial compared to those used in previous studies, because more complicated perception-action processes are required in daily life and sports training.

The participants conducted ten blocks with a short break (1 min between each block, 3 min between blocks five and six) from ML during Session 1 (initial ML: ML1-1 to ML1-10), Session 2 (overlearning: ML2-1 to ML2-10) and Session 3 (overlearning: ML3-1 to ML3-10). During Session 1, they also performed one block at 1 h and 4 h after the initial ML to assess early consolidation (Consoli-1 h and Consoli-4 h). Each block consisted of the same ten trials throughout. Task performance was assessed by taking the mean area of the deviation from the target black line in the ten trials per block. Skill acquisition during initial ML and overlearning were calculated by the ratio of final block to first block on each day, defined as “initial performance gain” and “overlearning gain”. Memory consolidation and retention were evaluated by the ratio of task performance in Consoli-1h and Consoli-4h to ML1-10 as “early consolidation”, the ratio of ML2-1 to ML1-10 as “late consolidation”, and the ratio of ML3-1 to ML1-10 as “retention”.

### 2.4. Electroencephalography (EEG) Recording and Analysis

Resting-state continuous EEG was recorded before ML1-1, immediately after ML1-10 and before Consoli-1h. EEG recordings with a notch filter of 50 Hz were conducted using a Brain Products amplifier system (Brain Products GmbH, Gilching, Germany) and Brain Vision Professional Recorder (Brain Products GmbH, Gilching, Germany). The resting-state recording duration was set to 3 min. During this period, the participants sat in a comfortable reclining position with their eyes open. They were instructed not to move their eyes or body and to avoid engaging in any specific mental activity. To ensure compliance with the experimental requirements, their behavior was monitored by an examiner. In addition, continuous EEG was recorded during ML: ML1-1, ML1-5, ML1-6 and ML1-10. Electrooculograms (EOGs) of the left eye were simultaneously recorded with EEG recordings to remove artifacts caused by eyeblinks.

Continuous data were recorded with a sampling rate of 2000 Hz from electrode position C3 (contralateral to performed hand), which referenced A1–A2 in both earlobes according to the 10–20 system. The ground electrode was set at Fpz. Electrode impedance was maintained below 5 KΩ. The EEG data were analyzed by using Brain Vision Professional Analyser 2 (Brain Products GmbH, Germany). EEG artifacts caused by eye blinks were removed by independent component analysis (ICA) with EOG data. Then, all data sets were filtered by a bandpass filter at 0.1–100 Hz.

For the frequency spectral analysis, the realigned EEG data were transformed into individual frequency power spectra by applying a fast Fourier transformation. The output of this procedure was the mean power for each frequency bin in the range of 1–100 Hz (with a 0.1 Hz frequency resolution). For each participant, the spectral power was averaged for the α (8–13.9 Hz), β (14–29.9 Hz), low-γ (30–45 Hz) and high-γ (60–80 Hz) frequency bands. Each EEG oscillation at the resting state and during ML were normalized to the baseline resting-state EEG oscillation (i.e., before ML1-1).

### 2.5. Questionnaires for Psychological Aspects

#### 2.5.1. Menstrual Distress Questionnaire (MDQ)

The Japanese version of the MDQ was used to evaluate the prevalence and severity of premenstrual symptomatology for each subject [57]. The MDQ consisted of 46 symptoms in eight categories: pain, concentration, behavioral change, autonomic reactions, water retention, negative affect, arousal, and control. The symptoms were assessed by assigning a score of 1 (no symptoms), 2 (minimal), 3 (mild), 4 (moderate), 5 (strong), or 6 (severe) to each of the 46 items across the eight categories. The MDQ was administered three times during the follicular, preovulatory and luteal phases with each participant to confirm intraindividual alteration. The sum of scores in each category was used to evaluate each symptom. In this study, the scores measured on Day 1, when the participants conducted the initial ML, were used for analysis.

#### 2.5.2. Multidimensional Assessment of Interoceptive Awareness (MAIA)

The MAIA was used to evaluate interoceptive awareness in each participant and was represented by a 32-item self-report questionnaire with eight scales [58]. The scales were assessed by eight domains: (1) noticing (awareness of uncomfortable, comfortable, and neutral body sensations), (2) not distraction (tendency not to ignore or distract oneself from sensations of pain or discomfort), (3) not worrying (tendency not to worry or experience emotional distress with sensations of pain or discomfort), (4) attention regulation (ability to sustain and control attention to body sensations), (5) emotional awareness (awareness of the connection between body sensations and emotional states), (6) self-regulation (ability to regulate distress by attention to body sensations), (7) body listening (active listening to the body for insight), and (8) trusting (experience of one’s body as safe and trustworthy). The items were answered on a 6-point Likert scale (0 to 5), with higher scores indicating higher interoceptive awareness. MAIA was administered once before starting the experiment in Session 1. The mean scores on each scale were used to analyze interoceptive awareness across groups.

### 2.6. Urine Samples

Partial urine samples of 10 mL were collected in the follicular, preovulatory and luteal phases irrespective of the assigned group to ensure that the participants were in the correct menstrual phase. The samples were collected in the morning and transported to the university where they were frozen at −20 °C. Further analysis was conducted by a clinical laboratory test company (SRL Inc., Kanagawa, Japan). Urinary sex steroid concentrations were assessed for pregnanediol-3-glucuronide (PdG), estradiol-2 (E2) and creatinine. PdG, the urinary metabolite of the luteal phase hormone progesterone [59], was quantified using gas chromatography-mass spectrometry (GC/MS, GCMS-QP2010, SHIMADZU Corp.). E2 was quantified by radioimmunoassay (RIA) ammonium sulfate precipitation using a liquid scintillation counter system (AccuLLEX LSC-8000). Creatinine (Cr) was evaluated by an enzyme method using a biochemical autoanalyzer (BioMajaesty JCA-BM8060, JCA-BM6010m, JEOL Ltd., Tokyo, Japan). Cr levels of all samples were determined for reporting steroid concentrations relative to this fundamental urine parameter. Therefore, PdG and E2 levels were evaluated by the ratio of each biomarker to the urinary Cr level as referenced in previous studies [60,61].

### 2.7. Statistical Analysis

Regarding baseline comparisons among the three groups (follicular, ovulation and luteal groups), participant physical characteristics, behavioral performance, and PMS and interoceptive awareness scores were entered into one-way ANOVA with ‘group’ as the between-subject factor.

To confirm the menstrual cycle in each participant, E2/Cr and PdG/Cr in each phase in all participants were entered into one-factor repeated measures ANOVA, with ‘phase’ (follicular, ovulation and luteal) as the within-subject factor. To ensure the particular phase in each group, E2/Cr and PdG/Cr in each measured phase were entered into one-way ANOVA, with ‘group’ as the between-subject factor.

Task performances during ML were entered into three-factor mixed-design ANOVA, with ‘block’ (thirty blocks in three days) and ‘session’ (Session 1, Session 2, Session 3) as the within-subject factors and ‘group’ as the between-subject factor. If a significant three-way interaction was observed, follow-up ANOVA by each factor was conducted with ‘group’ as the between-subject factor and ‘block’ as the within-subject factor. Additionally, in order to compare the performance gain on each day, the initial performance gain and overlearning gain were entered into one-way ANOVA with ‘day’ as the between-subject factor. Moreover, to compare final performance by overlearning, each day for the three groups the ratio of task performances in the final block of each session (ML2-10 and ML3-10) to the initial block (ML1-1) was entered into one-way ANOVA with ‘group’ as the between-subject factor.

Regarding memory consolidation after ML, the ratios of Consoli-1h, Consoli-4 h and ML2-1 to ML1-10 were entered into two-factor mixed-design ANOVA with ‘block’ (1 h and 4 h after ML during Session 1 and block 1 during Session 2) as the within-subject factor and ‘group’ as the between-subject factor.

Resting-state EEG oscillations were entered into two-factor mixed-design ANOVA with ‘block’ (baseline before ML1-1 and immediately and 1 h after ML1-10) as the within-subject factor and ‘group’ as the between-subject factor. To compare the EEG oscillations during ML among the groups, we conducted two-factor mixed-design ANOVA with ‘block’ (ML1-1, ML1-5, ML1-6, ML1-10) as the within-subject factor and ‘group’ as the between-subject factor.

To probe the factors influencing the online and offline effects of ML, a correlation analysis was conducted. We investigated the correlation between the online and offline effects of ML and the other parameters. Additionally, we performed correlation analyses between E2/Cr and PdG/Cr and EEG oscillations, PMS scores and interoceptive awareness scores to explore changes related to sex steroids.

In all analyses using repeated measures and mixed-design ANOVAs, the Greenhouse–Geisser correction was used to correct for non-sphericity if necessary, and Bonferroni’s post hoc tests were used for pairwise comparisons. A *p*-value of < 0.05 was considered statistically significant. Data were analyzed using a statistical software package (IBM SPSS Version 18, Chicago, IL, USA). All data are expressed as the mean ± SEM.

## 3. Results

### 3.1. Baseline Comparisons among Three Groups

Baseline comparisons among the three groups are shown in Table 1. For physical characteristics, the results of one-way ANOVA revealed no significant differences among the three groups in age, height, weight, or Edinburgh Handedness Inventory scores.

Regarding the behavioral data, we did not find significant differences among the three groups in maximal force and initial performance at ML1-1.

In regard to resting-state EEG oscillations, there was a significant main effect of ‘group’ on β oscillations, accompanied by a significant difference between the ovulation and luteal groups (*p* = 0.019), but no such effect on α oscillations or low or high γ oscillations.

For PMS scores evaluated by the MDQ, there were significant main effects of ‘group’ in the domains of pain, water retention, autonomic reactions, and negative affect but not in the domains of behavior, arousal, concentration, and control. The post hoc tests revealed significantly higher scores in the luteal group than in the other groups in terms of pain (ovulation: *p* = 0.008), water retention (follicular: *p* = 0.009, ovulation: *p* = 0.008), autonomic reactions (follicular: *p* = 0.02, ovulation: *p* = 0.003) and negative affect (follicular: *p* = 0.020, ovulation: *p* = 0.006).

Regarding interoceptive awareness scores assessed by the MAIA, there was a significant main effect of ‘group’ in the domain of body listening, with significantly lower scores in the luteal group than in the follicular group (*p* = 0.014), but not in the other domains: noticing, not-distracting, not-worrying, attention regulation, emotional awareness, self-regulation and trusting.

### 3.2. Sex Neurosteroids

**Figure 3:**
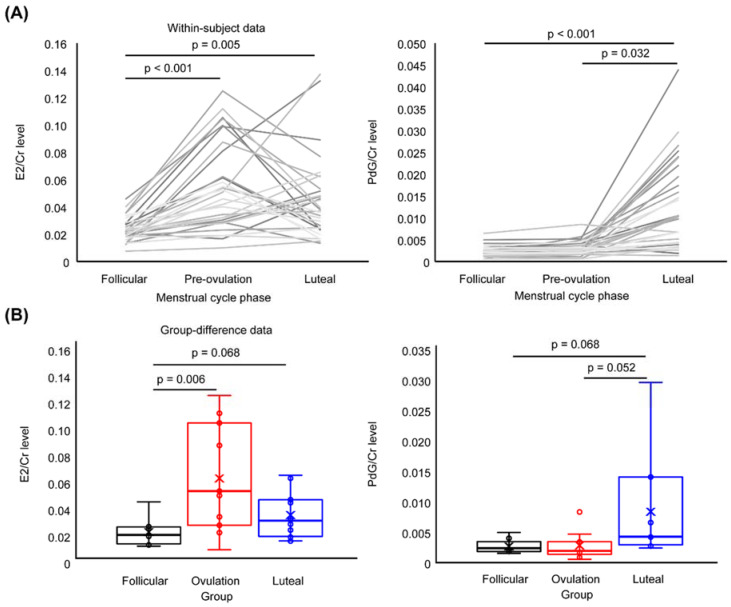
Sex neuro-steroid levels. (**A**) shows the fluctuations in E2/Cr (left panel) and PdG/Cr (right panel) levels across menstrual cycle phases in each participant (*n* = 31). E2/Cr levels increased in the preovulation and luteal phases relative to the follicular phase. The PdG/Cr level increased in the luteal phase compared to the other phases. (**B**) presents the group differences in E2/Cr (left panel) and PdG/Cr (right panel) on Day 1. E2/Cr was highest in the ovulation group, whereas PdG/Cr level was highest in the luteal group.

Figure 3A shows the within-subject fluctuations in sex neuro-steroids in all participants. One-factor repeated-measures ANOVA revealed a significant main effect of ‘phase’ (follicular, ovulation and luteal) on E2/Cr levels (F(2,60) = 11.350, *p* < 0.001) and PdG/Cr levels (F(2,60) = 28.542, *p* < 0.001). There were higher E2/Cr levels in the preovulatory (*p* < 0.001) and luteal (*p* = 0.005) phases than in the follicular phase. There were higher PdG/Cr levels in the luteal phase than in the follicular (*p* < 0.001) and preovulatory (*p* = 0.032) phases.

**Figure 4:**
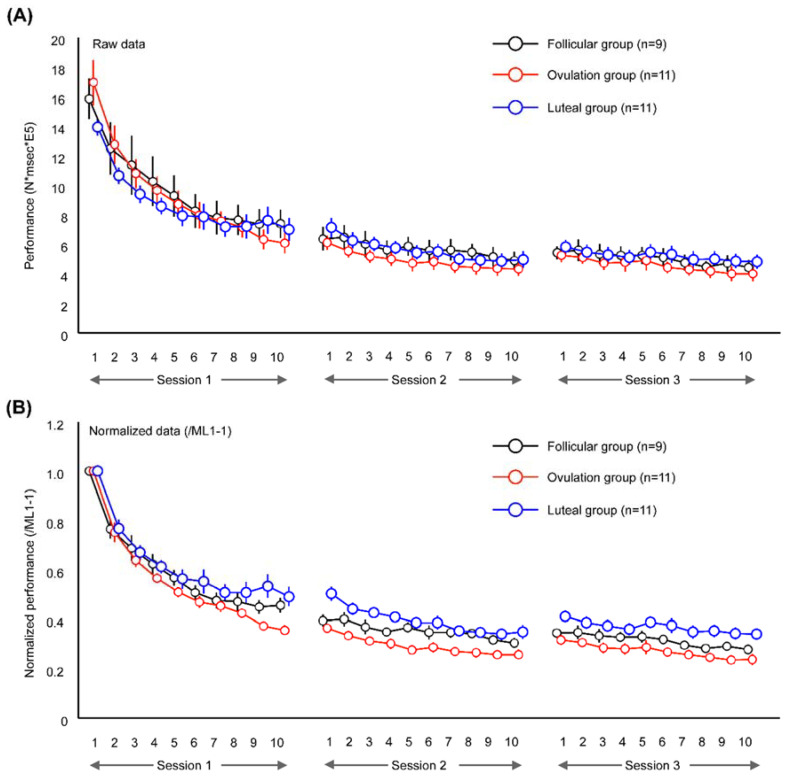
Changes in task performance during the present experiment. (**A**) shows the change in task performance presented as raw data. Task performance gradually decreased relative to the preceding ML trials in all groups, with significant improvement during Session 2 and Session 3 compared to Session 1. (**B**) presents task performance normalized to ML1-1.

Figure 4B presents group differences in sex neuro-steroids during Session 1. One-way ANOVA showed a significant main effect of ‘group’ on E2/Cr levels (F(2.28) = 6.250, *p* = 006) and PdG/Cr levels (F(2,58) = 4.149, *p* = 0.026). There were higher E2/Cr levels in the ovulation group than in the follicular group (*p* = 0.006) and a tendency towards higher E2/Cr levels in the luteal group than in the follicular group (*p* = 0.068). The PdG/Cr levels tended to be higher in the luteal group than in the follicular (*p* = 0.068) and ovulation (*p* = 0.052) groups.

### 3.3. Online Effect of ML

Figure 4A,B shows the changes in task performance throughout the present experiment. Three-factor mixed-design ANOVA revealed significant interactions of ‘session’ × ‘block’ × ‘group’ (F(36,504) = 2.620, *p* < 0.001, ηp^2^ = 0.173), ‘session’ × ‘block’ (F(18,36) = 75.709, *p* < 0.001, ηp^2^ = 0.730) and ‘block’ × ‘group’ (F(18,252) = 2.051, *p* = 0.008, ηp^2^ = 0.836) and main effects of ‘block’ (F(2.955,82.731) = 142.559, *p* < 0.001, ηp^2^ = 0.836) and ‘session’ (F(1.078,30.177) = 133.287, *p* < 0.001, ηp^2^ = 0.826), but there was no interaction of ‘session’ × ‘group’ (F(4,56) = 1.393, *p* = 0.248, ηp^2^ = 0.090) or main effect of ‘group’ (F(2.28) = 0.184, *p* = 0.883, ηp^2^ = 0.013). As there was a main effect of ‘session’, follow-up ANOVA day revealed a significant interaction of ‘block’ × ‘group’ only on Day 1 (F(18,252) = 2.949, *p* < 0.001, ηp^2^ = 0.174) and a main effect of ‘block’ (F(2.796,78.297) = 133.600, *p* < 0.001, ηp^2^ = 0.833); however, there were no effects during Sessions 2 and 3.

**Figure 5:**
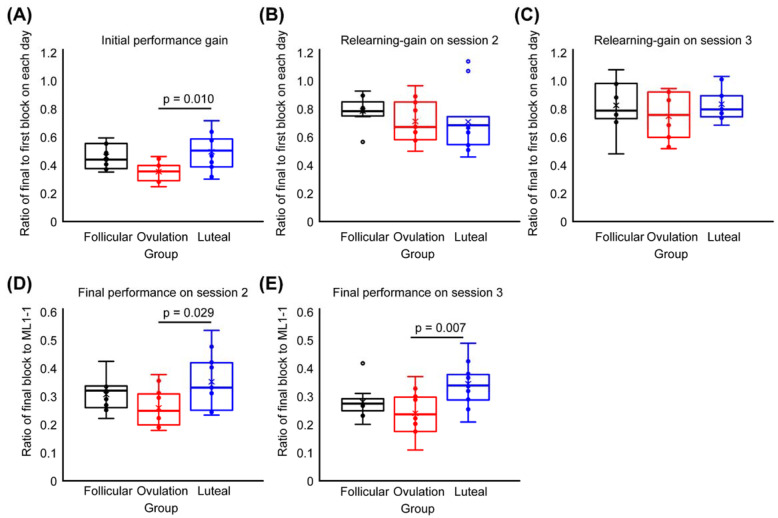
Performance gain during initial ML and overlearning and final performance on each day. (**A**) shows initial performance gain during Session 1. There was lower initial performance gain in the luteal group than in the ovulation group. (**B**,**C**) present the overlearning gain during Sessions 2 and 3; there were no differences among the three groups. (**D**,**E**) show significantly lower final performance in the luteal group than in the ovulation group during Sessions 2 and 3. These results indicated that the initial performance gain during Session 1 influenced task performance not only on the next day but also 1 week after the initial ML.

Regarding the group comparisons of performance gain, the performance gain by initial ML was significantly lower in the luteal group than in the ovulation group, as evidenced by the results from one-way ANOVA with a ‘group’ effect (F(2.28) = 5.395, *p* = 0.010) and a post hoc test (*p* = 0.011) (Figure 5A), while overlearning gain did not differ across the three groups during Session 2 or 3 (Figure 5B,C).

Additionally, the final performances on each day were significantly different among the three groups during Session 2 (F(2.28) = 3.855, *p* = 0.033, Figure 5D) and Session 3 (F(2.28) = 5.629, *p* = 0.009, Figure 5E). Post hoc tests revealed that the final performances during Sessions 2 and 3 were lower in the luteal group than in the ovulation group (*p* = 0.029, *p* = 0.007), similar to the findings from Session 1 (initial performance gain).

### 3.4. Offline Effect of ML

There were no significant differences in early and late consolidation among the three groups. This was supported by the results of two-factor mixed-design ANOVA, which showed no significant interaction of ‘group’ × ‘block’ (F(4,56) = 2.358, *p* = 0.064, ηp^2^ = 0.114), main effect of ‘group’ (F(2.28) = 1.800, *p* = 0.184, ηp^2^ = 0.114), and main effect of ‘block’ (F(2,56) = 1.427, *p* = 0.249, ηp^2^ = 0.049).

### 3.5. EEG Oscillatory Activity

Regarding resting-state oscillatory activity, there were no significant differences in brain oscillations in the α, β, and low and high γ bands among the three groups. Additionally, EEG oscillations during ML did not differ among the three groups.

### 3.6. Factors Influencing the Online and Offline Effects of ML

The amount of initial performance gain, shown by the ratio of performance at ML1-10 to ML1-1, was significantly associated with E2/Cr levels (r = −0.372, *p* = 0.039) and three domains of the MDQ (Table 2): water retention (r = 0.412, *p* = 0.021), concentration (r = 0.375, *p* = 0.037) and autonomic reactions (r = 0.464, *p* = 0.009).

Regarding the offline effects of ML, early consolidation, evaluated by the ratio of performance 4 h after ML (Consoli-4 h) to the final block during ML (ML1-10), was significantly associated with E2/Cr levels (r = 0.597, *p* < 0.000) and β oscillations during the late phase of ML (ML1-6: r = −0.389, *p* = 0.030; ML1-10: r = −0.492, *p* = 0.005). Additionally, early consolidation (1 h and 4 h after initial ML) was significantly related to the amount of initial performance gain during ML during Session 1 (1 h: r = −0.379, *p* = 0.035; 4 h: r = −0.551, *p* = 0.001).

Late consolidation, assessed by the ratio of performance in the first block during Session 2 (ML2-1) to the final block of ML during Session 1 (ML1-10), was significantly correlated with the amount of initial performance gain during Session 1 (r = −0.539, r = 0.002) and early consolidation during Session 1 (1 h: r = 0.395, *p* = 0.028; 4 h: r = 0.535, *p* = 0.002).

Retention, assessed by the ratio of performance in the first block during Session 3 (ML3-1) to the final block of ML during Session 1 (ML1-10), was significantly associated with the amount of initial performance gain (r = −0.575, r = 0.001) and late consolidation (r = 0.646, *p* < 0.000).

Regarding performance gain by overlearning, the amounts of initial performance gain and overlearning gain during Session 2 were significantly associated with the amount of overlearning gain during Session 3 (Session 1: r = 0.398, *p* = 0.027; Session 2: r = 0.408, *p* = 0.023).

### 3.7. Changes Related to Sex Steroids

E2/Cr was associated with noticing (r = 0.371, *p* = 0.040) and not-distraction (r = −0.386, *p* = 0.032) scores on the MAIA. Additionally, Preg/Cr was related to resting-state β oscillations (r = 0.387, *p* = 0.031), low-γ oscillations (r = 0.652, *p* < 0.001) and high-γ oscillations (r = 0.658, *p* < 0.001) immediately after ML during Session 1 and negative affect scores on the MDQ (r = 0.405, *p* = 0.024).

## 4. Discussion

To test our hypothesis that both the online and offline effects of ML would differ depending on the menstrual cycle, we compared the amount of performance gain by ML and overlearning, early and late consolidation, and final performance one week after ML among the follicular, ovulation and luteal groups. To explain the behavioral differences, we also examined M1 oscillations in the resting state and during initial ML as neurophysiological aspects and PMS and interoceptive awareness as psychological aspects.

As a result, novel findings, by comparison among the menstrual phase groups, are as follows.

(1) Performance gain in the initial ML during Session 1 depended on menstrual cycle; there was a lower initial performance gain in the luteal group than in the ovulation group. This behavioral difference could be explained by hyperexcitability in M1 and some symptoms of PMS in the luteal group.

(2) The offline effect of the initial ML did not differ across the menstrual cycle phases, supported by no difference in early and late consolidations. Despite not being related to menstrual cycle, higher early consolidation was observed in the participants with higher E2/Cr levels, higher β oscillations in the late phase of ML (ML1-6 and ML1-10) and lower initial performance gain. Additionally, higher early consolidation induced greater late consolidation assessed the following day.

(3) Final performances during Sessions 2 and 3, similar to Session 1 (initial performance gain, mentioned in main finding 1), were lower in the luteal group than in the ovulation group. The overlearning gain one week after the initial ML was superior in participants with a higher initial performance gain and overlearning gain in the late consolidation phase (Session 2).

### 4.1. Online Effect on ML across the Menstrual Cycle

The present group-comparison analysis revealed a lower online effect on ML in the luteal group than in the ovulation group. To our knowledge, this is the first study to show distinct online effects on ML across menstrual cycle phases, although menstrual-related differences in declarative learning and cognitive and motor performance are well known. There are a number of possible explanations for this finding. First, the sex neuro-steroids progesterone and estradiol could be one explanation for the lower online effect on ML in the luteal phase than in the preovulatory phase, as peripheral concentrations of progesterone and estradiol have been found to be well correlated with concentrations in the brain [62,63]. The fluctuations in these neuro-steroid levels can act on neurotransmitter systems to alter the balance of facilitation and inhibition. In animal and human studies, estradiol increased glutamate release and glutamate sensitivity of AMPA receptors via estradiol receptors (see review by Dachtler and Fox [64]), which modulate the balance of facilitation and inhibition in favor of facilitation [65]. Conversely, the major metabolite of progesterone, allopregnanolone, has been shown to upregulate the GABAergic system, leading to an increase in the inhibition of neuronal excitability [66]. Although we did not directly measure allopregnanolone levels, these are correlated with progesterone levels, particularly in the luteal phase [63]. Considering the fluctuation in urinary estradiol and progesterone levels across the menstrual cycle in the present study, these sex neuro-steroids were likely to influence the balance of facilitation and inhibition in M1. In a previous study examining cortical plastic changes induced by ML, M1 plastic changes and behavioral improvements in ML depended on a shift in the balance of the synaptic efficacy of horizontal interneural circuits towards less inhibition and more facilitation [67,68]. In the present study, resting-state β oscillation, which reflects neural inhibition related activity [38,39,40], in the luteal group have been significantly lower than in the ovulation group before ML, which might show abnormal hyperexcitability in M1 induced by the interaction effect of sex neuro-steroids progesterone and estradiol in luteal phase [46]. Considering that deficit of M1 inhibition impaired motor learning and neural plasticity [69,70], lower β oscillation might involve a lower online effect on ML in the luteal group. On the other hand, β oscillations during ML were not different across the menstrual cycle, which counted against our hypothesis, with a poor reduction in β oscillations during ML in the luteal group. This might be why the EEG signal mainly reflects excitatory pyramidal neural activity in the cerebral cortex instead of horizontal interneural circuits. Considering the observed correlation between E2/Cr and initial performance gain, although it is possible that progesterone could have interfered with the facilitatory effect of estrogen on the initial performance gain, the present study could not prove a clear reason for the poor online effect on ML in the luteal phase. Therefore, further study examining changes in horizontal interneural activity by ML across the menstrual cycle is required.

PMS-related abnormal M1 excitability could be another explanation involved in the lower online effect on ML in the luteal group. In the luteal phase, a majority of females experienced at least some degree of disharmony of mind and body, which is commonly termed PMS. This condition regularly recurs with diverse nonspecific physical, emotional, behavioral, and cognitive symptoms, which usually abate shortly after the onset of menses [71,72]. The present study also showed worse physical (pain and water retention) and emotional (autonomic reactions and negative affect) conditions in the luteal phase based on the results of the MDQ. This assumption could also be explained by PMS-related abnormal M1 excitability. Although higher intracortical inhibition in M1 was observed in the luteal phase due to progesterone action [47], disinhibition and/or hyperexcitation were observed in the participants with PMS [46]. Although we did not directly measure the inhibitory circuit, resting-state β oscillations at baseline represented an inhibitory function, significantly decreased in the luteal group compared with the other groups, against progesterone-dependent increased inhibition. Therefore, the present results indicate that PMS might be a possible explanation for impaired performance gain during initial ML in the luteal phase. However, further study focused on the effect of PMS is needed because the functional and anatomical alteration induced by PMS remains unclear.

### 4.2. Offline Effect on ML across the Menstrual Cycle

The present group-comparison analysis showed that early and late consolidation after ML did not differ across the menstrual cycle phase against our hypothesis that early consolidation would be facilitated by progesterone-induced higher β oscillations in the late phase of ML. One explanation for this result could be attributed to the lack of differences in β oscillations during ML across the menstrual cycle phases. M1 oscillations have a crucial role in early consolidation after ML [36,37]. Notably, increased β oscillations in the late phase induced robust early consolidation of skill acquisition after ML [36]. Studies investigating neurophysiological brain mechanisms underlying motor control have shown increased β oscillation-induced inhibitory function within neuronal motor control networks [73,74], which seems to be relevant to the maintenance of the current motor state [38]. These findings support the notion that higher β oscillations were involved in functional reorganization associated with ML and early consolidation [75]. In the present study, as we explored the relation between β oscillations during ML and the offline effect on ML, increased β oscillations in the late phase of ML are likely to have facilitated early consolidation after ML, although this was not related to the menstrual cycle. These results were supported by a previous study showing that tACS at β frequency interacts with motor-cortical β oscillations, reflecting functional reorganization over the time course of ML [76]. However, these correlations with β oscillations did not expand to late consolidation at 24 h after ML and retention six days after the initial ML. This could be due to distinct neural mechanisms involved in early consolidation, which occurs within minutes to hours, and late consolidation and retention, which is observed after durations of days and more. However, further research is needed because the present study could not examine in detail the differences in neural mechanism between early and late consolidation or retention.

### 4.3. Overlearning Effect and Final Performance across the Menstrual Cycle

The overlearning gain one day and one week after initial ML (Session 2 and Session 3) did not differ across the menstrual cycle in contrast to initial learning (Session 1). Interestingly, overlearning gain during Session 3 depended on initial performance gain and overlearning gain during Session 2, although it was not related to the menstrual cycle. This relation may suggest that successive performance in the initial learning phase and overlearning in the late consolidation phase benefited overlearning over a long interval during Session 3 in the transfer to long-term memory. Our assumption was supported by the retrieval effort hypothesis (REH), confirmed in declarative memory processing [77], which indicated that successful retrieval practice benefited memory most when successful retrieval was more effortful rather than less effortful. That is, the memory gains during (over)learning favor superior overlearning because tasks with proportionally higher criteria are successfully recalled with greater effort rather than with less effort. Therefore, the present study might indicate that overlearning gain with a long interval may depend on initial performance gain, although further studies need to examine whether REH may adapt not only to declarative learning but also procedural learning. Moreover, considering that the difference in the initial performance gain during Session 1 was maintained until the final performance, as shown by poorer performance during Session 2 and Session 3 in the luteal group relative to the ovulation group, the question of when to start learning new sensorimotor skills could be very important in females regarding obtaining the expected gains.

### 4.4. Limitations

The present study has several limitations when clarifying menstrual cycle-dependent differences in procedural memory induced by ML. First, the sample size was small. In the present study, we recruited 41 females with informed consent based on the results of power analyses to determine the recommended sample size, which indicated that more than 30 total participants would be needed to detect an effect size of 0.25, with the α set at 0.05 and β set at 0.20, but 10 participants could not finish all experiments for several reasons. To obtain more robust evidence, we should retest the present results in many participants. Second, the present study was designed as a between-group comparison. The best way to compare behavior across the menstrual cycle phase is a within-subject study. However, it was difficult to adopt this methodology because of the potential effect of prior ML, as shown. Third, the present study was not conducted in the context of a double-blind design. Participants were aware of when they were conducting initial ML during the menstrual cycle phase, although the measured cycle was blinded in examiners. Fourth, the ML used might include two motor learning components, e.g., “adapted” and “sequence” motor learning [51], but we only used the same trials for complex motor task for ML. Additionally, we cannot state definitively whether the participants were aware that the trials were repeated in each block. Therefore, further studies are needed to understand menstrual cycle-dependent differences in ML and procedural memory.

## 5. Conclusions

The present study indicated that performance gain in initial ML differs across the menstrual cycle; it is lower in the luteal phase and higher in the preovulation phase due to psychological aspects, and the difference was not explained by alteration of M1 oscillations. Additionally, these distinct performance gains influenced not only overlearning one week after the initial ML but also the final performance achieved by overlearning.

## Figures and Tables

**Table 1:** Baseline assessments of the three groups.

	Follicular	Ovulation	Luteal	F [df, Error]	*p*
Physical characteristics					
Age (y.o.)	19.67 ± 0.33	20.18 ± 0.3	20.09 ± 0.42	0.557 (2.28)	0.579
Height (cm)	160.81 ± 1.75	161.71 ± 2.23	160.44 ± 1.06	0.147 (2.28)	0.864
Weight (kg)	55.19 ± 1.65	57.51 ± 3.45	58.17 ± 2.62	0.293 (2.28)	0.748
Edinburgh Handedness Inventory (points)	100 ± 0	96.97 ± 2.17	98.7 ± 1.3	0.926 (2.28)	0.408
Behavioral data					
Maximal force (N)	49.63 ± 4.13	52.82 ± 4.19	56.38 ± 2.38	0.841 (2.28)	0.442
Initial performance (N*sec*E5)	15.85 ± 1.36	16.95 ± 1.522	13.96 ± 0.59	1.646 (2.28)	0.211
Resting-state EEG oscillations					
α oscillations (µV^2^/Hz)	29.15 ± 8.42	27.64 ± 5.4	16.13 ± 10.57	1.584 (2.28)	0.224
β oscillations (µV^2^/Hz)	4.22 ± 0.54	4.87 ± 0.8	2.5 ± 0.23 *	4.642 (2.28)	0.018
Low γ oscillations (µV^2^/Hz)	1.05 ± 0.11	1.33 ± 0.24	0.77 ± 0.1	2.983 (2.28)	0.067
High γ oscillations (µV^2^/Hz)	0.43 ± 0.08	0.37 ± 0.08	0.26 ± 0.04	1.474 (2.28)	0.246
Menstrual Distress Questionnaire (MDQ) (points)					
Pain	8.89 ± 2.3	6.55 ± 1.82	15.36 ± 1.13 †	5.727 (2.28)	0.008
Behavioral change	8.89 ± 1.72	7.09 ± 2.33	14.73 ± 9.78	2.746 (2.28)	0.081
Water retention	5.44 ± 1.14	4.82 ± 1.21	11.55 ± 1.46 *	8539 (2.28)	0.001
Arousal	8.89 ± 1.5	7.82 ± 1.7	6.91 ± 1.18	0.430 (2.28)	0.655
Concentration	8.67 ± 2.83	5.91 ± 2.48	13.91 ± 2.9	2.324 (2.28)	0.116
Autonomic reactions	1.78 ± 0.76	1.18 ± 0.62	5.09 ± 0.93 *	7.533 (2.28)	0.002
Negative affect	7.56 ± 2.1	6.27 ± 2.99	19.73 ± 3.16 *	6.922 (2.28)	0.004
Control	2.33 ± 1.12	0.82 ± 0.54	3 ± 0.95	1.726 (2.28)	0.196
Multidimensional Assessment of Interoceptive Awareness (MAIA) (points)					
Noticing	2.81 ± 0.22	2.73 ± 0.27	2.77 ± 0.33	0.019 (2.28)	0.981
Not-distracting	3.59 ± 0.33	2.91 ± 0.22	3.24 ± 0.27	1.554 (2.28)	0.229
Not-worrying	2.56 ± 0.28	2.52 ± 0.2	2.7 ± 0.19	0.196 (2.28)	0.823
Attention regulation	2.81 ± 0.15	2.57 ± 0.24	2.74 ± 0.28	0.261 (2.28)	0.772
Emotional awareness	3.27 ± 0.33	2.53 ± 0.26	2.52 ± 0.28	2.068 (2.28)	0.145
Self-regulation	2.18 ± 0.18	1.8 ± 0.2	2.06 ± 0.26	0.759 (2.28)	0.477
Body listening	2.85 ± 0.26	2.18 ± 0.18	1.9 ± 0.21 ‡	4.908 (2.28)	0.015
Trusting	3.07 ± 0.26	2.61 ± 0.31	2.64 ± 0.32	0.690 (2.28)	0.51

Note. The asterisk (*) shows a significant difference compared to the follicular and ovulation groups. The dagger (†) indicates a significant difference compared to the ovulation group. The double dagger (‡) indicates a significant difference compared to the follicular group.

**Table 2:** Relationship between the initial ML performance gain and PMS.

MDQ Domains	r	*p*
Pain	0.317	0.082
Behavioral change	0.348	0.055
Water retention	0.412	0.021 *
Arousal	−0.008	0.966
Concentration	0.375	0.037 *
Autonomic reactions	0.464	0.009 *
Negative affect	0.299	0.102
Control	0.098	0.602

Note. The asterisk (*) shows a significant correlation between the online effect of initial ML and PMS condition.
